# Comparative performance analysis of lead-free perovskite solar cells based on FASnI_3_, CsSnI_3_, KGeCl_3_, and CsGeI_3_ absorbers with optimized charge transport layers

**DOI:** 10.1039/d6ra02463g

**Published:** 2026-08-03

**Authors:** Zhongjie Wang, Yuhao Wang, Benxiong Hu, Chenxi Zheng, Jian Qu, Yunxiang Zhang, Qinfang Zhang

**Affiliations:** a School of Materials Science and Engineering, Yancheng Institute of Technology Yancheng 224051 PR China chzyx123@163.com chzyx123@gmail.com qfangzhang@gmail.com; b School of Art and Design, Yancheng Polytechnic College Yancheng 224005 PR China

## Abstract

Perovskite solar cells (PSCs) are promising for next-generation photovoltaics, but their commercial viability is hindered by the toxicity of lead (Pb). This study investigates the performance of lead-free PSCs by comparatively evaluating four different perovskite absorber layers: FASnI_3_, CsSnI_3,_ KGeCl_3_ and CsGeI_3_. Using SCAPS-1D numerical simulations, the impact of the absorber material, along with the thickness and carrier concentration of the electron and hole transport layers (ETL and HTL), on photovoltaic parameters was systematically analyzed. Results show that among the four materials, FASnI_3_ delivers the highest overall power conversion efficiency (PCE), followed by CsSnI_3_ and KGeCl_3_, while CsGeI_3_ exhibits the lowest. Subsequently, detailed optimization of FASnI_3_-based PSCs reveals the crucial and complex interplay between ETL (TiO_2_) and HTL (Spiro-OMeTAD) parameters (thickness, carrier concentration, and defect density) on device performance. A key finding is that the defect density in the ETL has a more significant adverse impact on PCE compared to the HTL, primarily by enhancing non-radiative recombination. This study provides vital insights and optimization strategies for developing high-efficiency, environmentally friendly lead-free perovskite solar cells.

## Abstract 2

Comparative evaluation of four lead-free perovskites finds FASnI_3_ best (PCE ∼ 16.7%). Optimizing TiO_2_ ETL (thickness, doping, defect density) boosts PCE to 19.3%, with ETL quality crucially outperforming HTL optimization.

## Graphical abstract

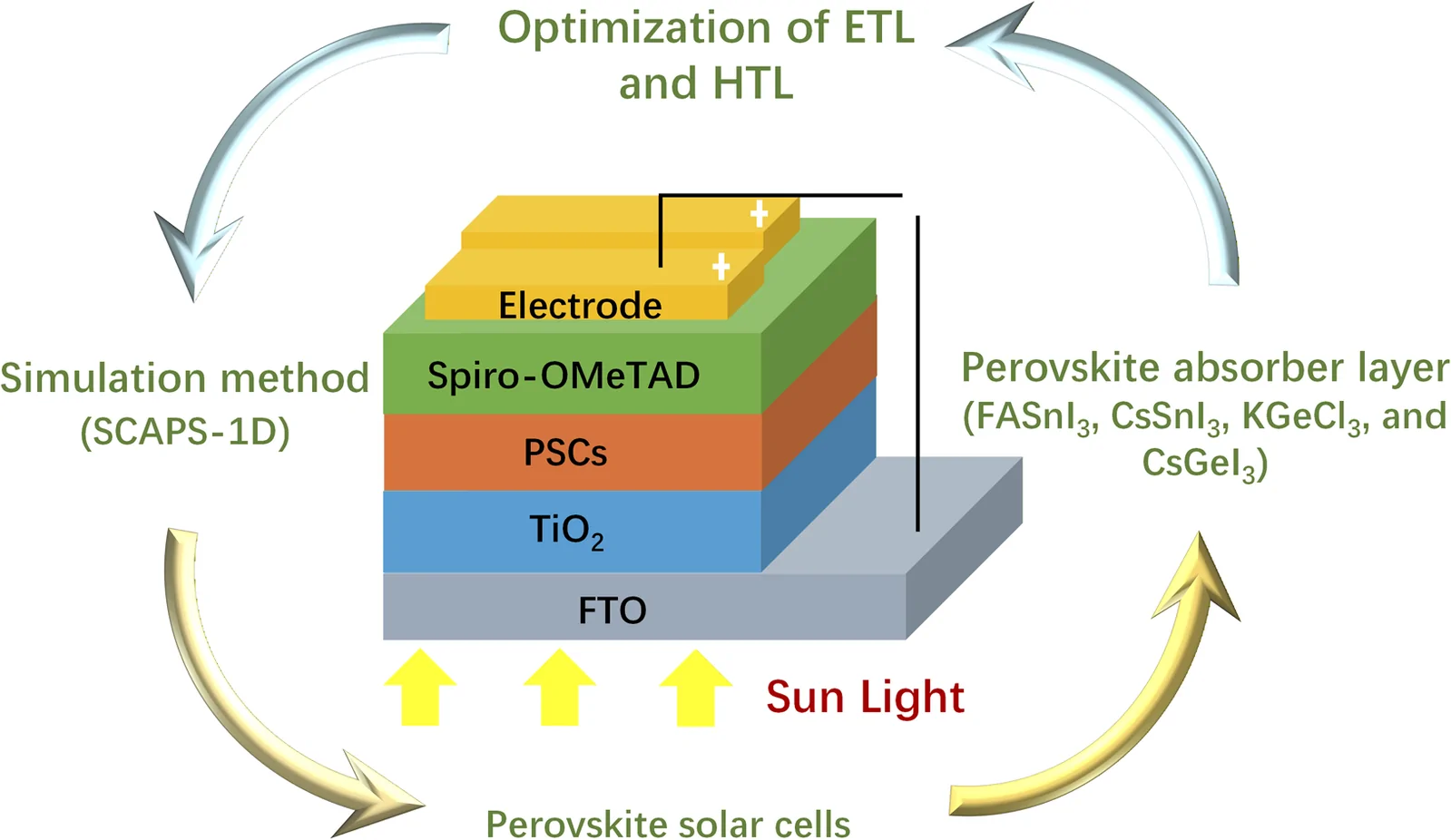

## 1. Introduction

As economic growth and environmental pressures intensify, the finite reserves and pollution issues of traditional fossil fuels have become increasingly prominent, prompting a global shift toward clean and sustainable new energy systems. In this transition, solar energy has emerged as a core pillar of sustainable development, thanks to its virtually limitless resource availability, zero carbon emissions, and broad geographical applicability.^1–4^ At present, the main application scenarios of solar energy include solar photovoltaic technology,^5–8^ photocatalytic reactions,^9–12^ photothermal reactions and other directions.^13–17^ Among them, solar photovoltaic technology has the advantages of high-efficiency energy conversion, low environmental impact, and flexible and diverse application scenarios.^18–20^ Especially the thin-film solar cell technology, with its lightweight, flexible and benders, excellent low-light performance, and significant cost-effectiveness, has attracted much attention and become one of the key areas promoting the popularization and innovation of solar energy applications.^21–23^

Among various solar technologies, perovskite solar cells (PSCs) emerge as a highly promising solution.^24–29^ This stems from their remarkable photoelectric conversion efficiency, which has surged from 3.8% to over 27% within just over a decade, now nearing the performance level of traditional crystalline silicon cells.^30,31^ Furthermore, PSCs offer distinct advantages in fabrication: they are relatively easy to prepare, can be processed at low temperatures, and enable significant production cost reductions through solution-based techniques using earth-abundant precursors for key components (*e.g.*, carbon, tin, iodine).^18,32^ Simultaneously, perovskite materials exhibit lightweight and flexible properties, enabling their integration into diverse applications such as building-integrated photovoltaics (BIPV), portable electronic devices, and space-based energy systems.^33^ Additionally, they demonstrate substantial potential for efficiency enhancement *via* tandem stacking with silicon-based solar cells, opening avenues for next-generation high-performance photovoltaic systems.^34,35^

Currently, although lead-based PSCs have high efficiency, the toxicity of lead poses a threat to the environment and human health, which hinders their large-scale application.^36–38^ Non-lead materials (such as tin-based perovskites) significantly reduce ecological risks by replacing lead ions, promoting the development of green energy. Moreover, these lead-free materials maintain good performance under low-light conditions, making them suitable for portable devices and building-integrated scenarios. Although their power conversion efficiencies are generally lower than those of lead-based counterparts when using pristine materials, recent advances in additive engineering, compositional tuning, and interface passivation have substantially improved their performance. Their environmentally benign nature and ongoing efficiency improvements continue to open new paths for sustainable photovoltaic technology, making them a key direction for future clean energy.^39,40^

Moreover, these materials still maintain excellent performance under low-light conditions, making them suitable for portable devices and integrated scenarios in buildings. Although their efficiency is slightly lower, their environmental characteristics and potential open up new paths for photovoltaic technology, contribute to the sustainable energy transition, and are a key direction for future clean energy. In 2023, Zihao Zhu and his team introduced trimethylthiourea (3T) as a bifunctional ligand during the spin coating of formamidinium tin triiodide (FASnI_3_) films to enhance film quality by promoting grain spreading and joining.^41^ This approach significantly improved the morphology and texture of the films, leading to record charge-carrier lifetime (123 ns), open-circuit voltage (0.92 V), and power conversion efficiency (14.0%), while ensuring excellent stability against humid air. In 2024, Tianjun Ma *et al.* introduced a multidentate chelating additive ACPC into Sn–Pb perovskite precursor, which anchored I^−^*via* hydrogen bonds and coordinated with Sn^2+^*via* carbonyl groups. This approach effectively suppressed I^−^ migration and Sn^2+^ oxidation, achieving a champion device with a PCE of 23.09% and a high open-circuit voltage of 0.902 V.^42^ In 2025, Rossyaila Matsna Muslimawati and his colleagues evaluated the potential of FASnI_3_ and cesium tin triiodide (CsSnI_3_) as the active layer of PSCs.^43^ In 2024, Nikhil Shrivastav *et al.* employed machine learning models combined with SCAPS simulation to explore KGeCl_3_ perovskite solar cells, optimizing absorber layer thickness, doping, and defect density. Their optimized device achieved a PCE of 19.62%, demonstrating an efficient approach for the development of PSCs.^44^ Therefore, the exploration of lead-free perovskite absorber materials has become an important research topic in the field of PSCs.

It is also important to address the stability of Sn-based and Ge-based perovskites relative to their Pb-based counterparts, as stability is a critical factor for practical applications. Experimentally, Pb-based perovskites (*e.g.*, MAPbI_3_, FAPbI_3_) suffer from moisture-induced degradation and thermal instability, but they are relatively resistant to oxidation because Pb exists in the stable +2 oxidation state. In contrast, Sn-based perovskites (*e.g.*, FASnI_3_, CsSnI_3_) face a more severe challenge: Sn^2+^ is easily oxidized to Sn^4+^ upon exposure to ambient oxygen and moisture, leading to rapid film degradation, p-type self-doping, and reduced device lifetime.^39,40^ Various strategies such as the addition of reducing agents (*e.g.*, SnF_2_, hydrazine derivatives) or encapsulation have been shown to mitigate this issue, as demonstrated by Zhu *et al.*^41^ who achieved improved stability with trimethylthiourea. Ge-based perovskites (*e.g.*, CsGeI_3_, KGeCl_3_) are intrinsically more stable against oxidation than Sn-based ones because Ge^2+^ has a higher oxidation potential. However, Ge^2+^ can still disproportionate into Ge^0^ and Ge^4+^ under certain conditions, and Ge-based perovskites tend to have poor film morphology and higher defect densities, which indirectly affect their operational stability.^44^ Overall, while Pb-based perovskites currently offer better environmental stability, the rapid progress in encapsulation and additive engineering for Sn-based and Ge-based materials is narrowing this gap.

Therefore, in order to promote the development of lead-free PSCs, this paper mainly investigates the effects of four types of lead-free perovskite materials on the performance of PSCs, including FASnI_3_, CsSnI_3_, KGeCl_3_, and CsGeI_3_. Firstly, the influence of the active layers on the performance of PSCs was systematically compared. The results showed that FASnI_3_, as the lead-free activation layer material for PSCs, achieved the highest device performance. Subsequently, the influence laws of thickness and carrier concentration on the performance of FASnI_3_-based solar cells were evaluated. It has been found that a higher doping concentration is beneficial for increasing the open-circuit voltage *V*_OC_, thereby enhancing the efficiency of PSCs.

## 2. Device modeling and materials parameters

### 2.1. Device modeling

**Fig. 1:**
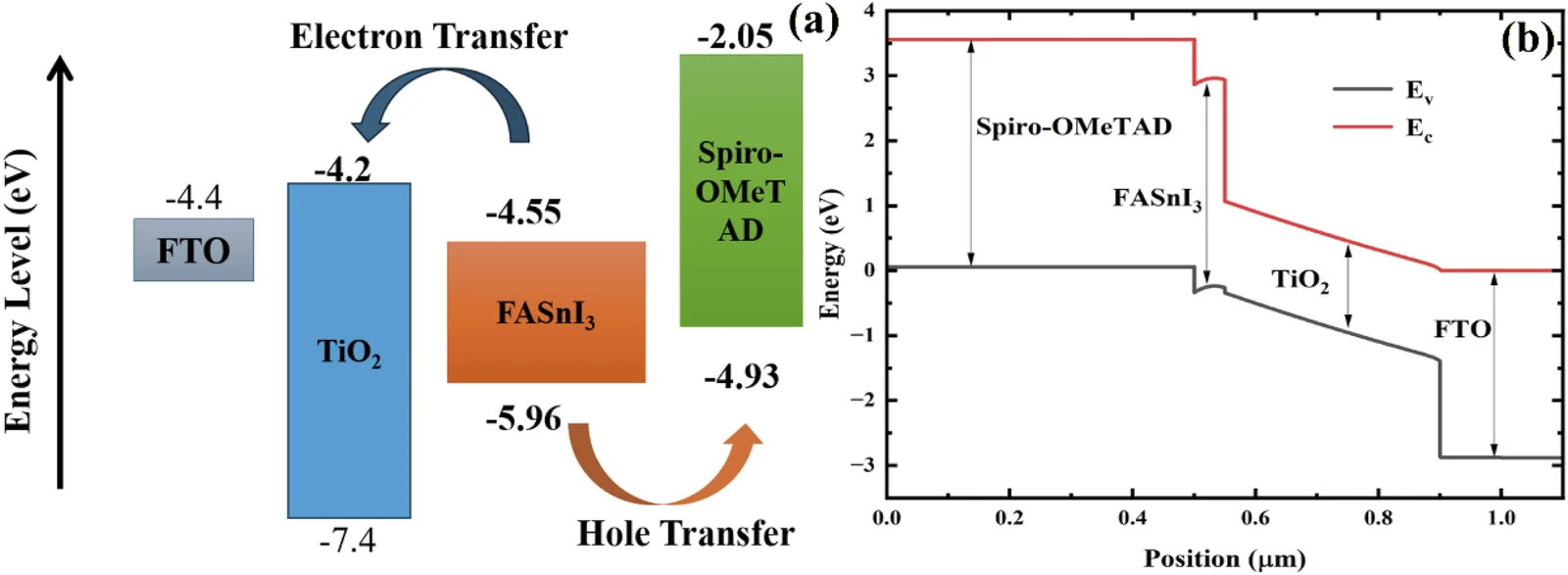
(a) Charge transfer and (b) band gap structure of the simulated PSCs.

In this numerical study, the SCAPS-1D software was employed to simulate the impact of perovskite (PVSK) layer materials in the FTO/TiO_2_/PVSK/Spiro-OMeTAD/Au structure.^38,45^ The front contact layer of PSCs is made of fluorine-doped tin oxide (FTO), which has high transparency and excellent conductivity. The electron transport layer uses TiO_2_ material with a thickness of approximately 50 nm to facilitate the efficient extraction and transmission of electrons. The PVSK absorber layer is the main light capture component. In this simulation, the four main types of contrastive perovskite materials are FASnI_3_, CsSnI_3_, KGeCl_3_, and CsGeI_3_. Under AM 1.5G illumination (100 mW cm^−2^) (following the Lambert–Bear optical model), electron–hole pairs are generated according to the Raman-Bear optical model. The hole transport layer Spiro-OMeTAD can achieve effective hole collection and transmission to the gold back contact layer. As shown in Fig. 1(a), under the action of the internal electric field (IEF), photogenerated electrons (e^−^) migrate to the electron transfer layer (ETL) of TiO_2_, while holes (h^+^) move to the hole transfer layer (HTL) of Spiro-OMeTAD. The energy bandgap arrangement of PSCs is shown in Fig. 1(b). Au was selected as the back contact due to its favorable work function alignment with Spiro-OMeTAD, excellent chemical stability, and widespread use as a benchmark in PSC simulations, enabling direct comparison with previous studies. Alternative metals such as Ag, Cu, or Al could also be explored as back contacts in future work. The regular (n-i-p) configuration (FTO/TiO_2_/perovskite/Spiro-OMeTAD/Au) was selected for this study to enable direct comparison with the key lead-free perovskite simulation works. This configuration is also well established in SCAPS-1D simulations, with reliable material parameters readily available in the literature.^46,47^

The performance characteristics of semiconductor devices, as modeled by the SCAPS simulation tool, are obtained through the solution of three core governing equations: the electron transport continuity equation, Poisson's equation, and the hole transport continuity equation. These equations are mathematically expressed below:^48–50^1
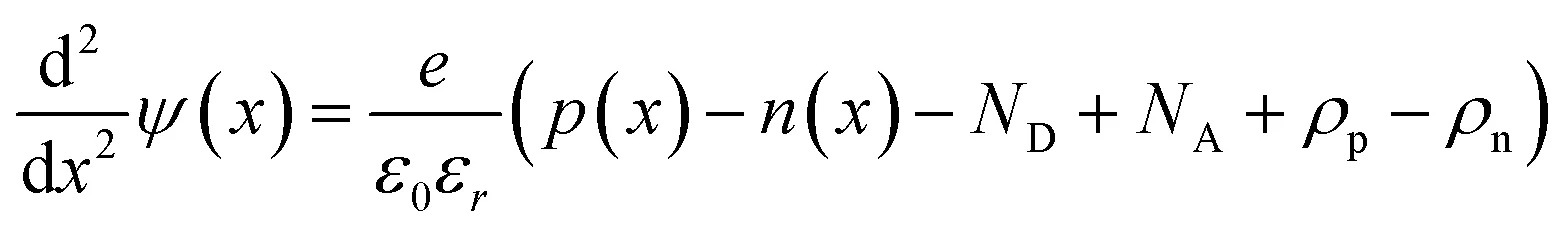
here, *ψ*(*x*) represents the electrostatic potential, while *ε*_0_ and *ε*_r_ denote the vacuum permittivity and material relative permittivity, respectively. The variables *p*(*x*) and *n*(*x*) correspond to hole and electron carrier concentrations, *N*_D_ and *N*_A_ to donor and acceptor impurity densities, and *ρ*_p_ and *ρ*_n_ to spatial charge distributions of holes and electrons. The elementary charge *e* quantifies charge magnitude.^50,51^2
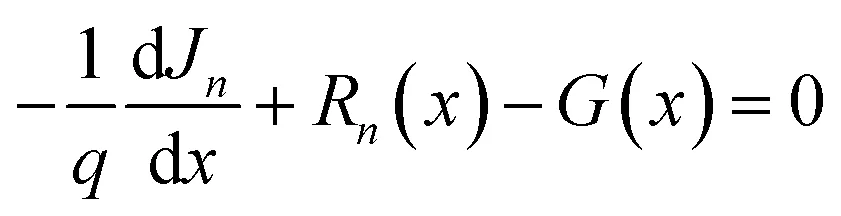


This equation captures the balance between charge generation (*G*) and recombination (*R*) for electrons, with *J*_*n*_ representing the electron current density.

Hole continuity equation:3
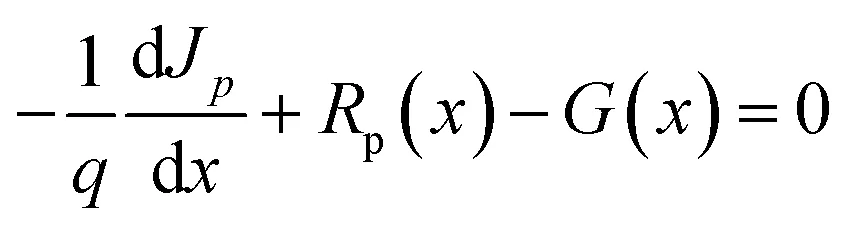


Similarly, this equation describes hole carrier dynamics, where *J*_p_ is the hole current density, and *G* and *R* again denote generation and recombination rates. In summary, these equations collectively describe the electrostatic potential distribution (*ψ*), charge carrier behavior, and doping effects (*N*_D_ and *N*_A_) in semiconductor materials. The interplay of these factors determines the electrical performance of device, including charge transport and recombination phenomena.

### 2.2. Device simulation

The baseline physical properties of PSCs (FTO, TiO_2_, FASnI_3_, and Spiro-OMeTAD), such as film thickness, energy band gap, carrier mobility, doping concentration, and defect density, are documented in Table 1, with parameters taken from refs. 52–54 It is worth noting that the conduction band offset between TiO_2_ (*χ* = 3.6 eV) and FASnI_3_ (*χ* = 3.52 eV) is Δ*E*_C_ = +0.08 eV. This small positive offset does not create a significant energy barrier for electron transfer from the perovskite to the ETL. Instead, such a minimal spike can effectively prevent electron backflow while allowing efficient forward electron extraction, thus having no detrimental impact on electron movement or overall device performance.

**Table 1:** Simulation parameters of PSCs in this work

	FTO	TiO_2_	FASnI_3_	Spiro-OMeTAD
*t* (nm)	500	50	300	200
*E*_g_ (eV)	3.5	3.2	1.4	3
*χ* (eV)	4	3.6	3.52	2.2
*ε*_r_	9	9	8.2	3
*N*_C_ (cm^−3^)	2.2 × 10^18^	2.0 × 10^18^	1 × 10^19^	2.2 × 10^18^
*N*_V_ (cm^−3^)	1.8 × 10^19^	1.8 × 10^19^	1 × 10^19^	1.8 × 10^19^
*µ*_n_ (cm^2^ V^−1^ s^−1^)	20	20	22	2.1 × 10^−3^
*µ*_p_ (cm^2^ V^−1^ s^−1^)	10	10	22	2.1 × 10^−3^
*N*_D_ (cm^−3^)	2.0 × 10^19^	9 × 10^16^	0	0
*N*_A_ (cm^−3^)	0	0	1.0 × 10^19^	1.8 × 10^19^
*N*_t_ (cm^−3^)	1.0 × 10^15^	1.0 × 10^15^	1.0 × 10^15^	1.0 × 10^15^

TiO_2_ was selected as the electron transport layer (ETL) in this study for several well-established reasons. First, TiO_2_ is one of the most widely used ETL materials in both experimental and simulation studies of perovskite solar cells due to its suitable conduction band edge (≈4.0 eV below vacuum), which aligns favorably with the electron affinity of FASnI_3_ (3.3 eV), facilitating efficient electron extraction while blocking holes. Second, TiO_2_ exhibits high transparency in the visible range, minimal parasitic absorption, and good chemical stability under ambient conditions. Third, the SCAPS-1D material parameters for TiO_2_ are well documented in the literature, allowing reliable benchmarking and comparison with previous simulation works.^45,55–57^ Although alternative ETLs such as SnO_2_ or ZnO may offer higher electron mobility, TiO_2_ remains a standard reference material for comparative device optimization, and our conclusions regarding the trends of thickness and doping effects are expected to be qualitatively transferable to other ETLs.

Spiro-OMeTAD was selected as the hole transport layer (HTL) for several well-justified reasons. First, Spiro-OMeTAD is the most widely used organic HTL in both experimental and simulation studies of perovskite solar cells, providing a well-established benchmark for comparative device optimization. Its highest occupied molecular orbital (HOMO) level (≈−5.2 eV) aligns appropriately with the valence band of FASnI_3_ (≈−5.0 eV, calculated from electron affinity and bandgap), facilitating efficient hole extraction while blocking electrons. Second, the material parameters of Spiro-OMeTAD (thickness, doping, mobility, defect density) are thoroughly documented in the SCAPS-1D literature, enabling reliable calibration and direct comparison with previous simulation works.^29,38,58^ Third, although inorganic HTLs such as CuI, CuSCN, NiO_*x*_, or MoO_3_ offer higher hole mobility and better thermal stability, their energy level alignment with lead-free perovskites (especially tin-based ones) is often less optimal, leading to increased interface recombination. Moreover, the choice of Spiro-OMeTAD in this study does not imply that it is the best possible HTL; rather, it serves as a representative and well-characterized reference material. The primary goal of this work is to compare four lead-free absorbers under identical transport layer conditions, and using a standard HTL ensures that the observed performance differences are attributed to the absorber materials themselves rather than to variations in HTL properties. Future optimization studies could explore inorganic HTLs for FASnI_3_-based devices, as suggested by the reviewer.

Table 2 presents the parameter sets for the four perovskite absorber layers (FASnI_3_, CsSnI_3_, KGeCl_3_, and CsGeI_3_), including band gap, electron affinity, dielectric constant, and carrier transport properties. The parameters for PSCs are from previous works.^59–61^

**Table 2:** Different simulation parameters in the perovskite layers

	FASnI_3_	CsSnI_3_	KGeCl_3_	CsGeI_3_
*t* (nm)	300	300	300	300
*E*_*g*_ (eV)	1.4	1.3	1.49	1.6
*χ* (eV)	3.52	3.6	3.52	3.6
*ε*_r_	8.2	9.93	3.52	18
*N*_C_ (cm^−3^)	1 × 10^19^	1.0 × 10^18^	1.0 × 10^18^	1.0 × 10^18^
*N*_V_ (cm^−3^)	1 × 10^19^	1.0 × 10^19^	1.0 × 10^19^	1.0 × 10^18^
*µ*_n_ (cm^2^ V^−1^ s^−1^)	22	1500	20	20
*µ*_p_ (cm^2^ V^−1^ s^−1^)	22	25	20	20
*N*_D_ (cm^−3^)	0	0	0	0
*N*_A_ (cm^−3^)	1.0 × 10^16–19^	1.0 × 10^16^	1.0 × 10^15^	1.0 × 10^15^
*N*_t_ (cm^−3^)	1.0 × 10^15^	1.0 × 10^15^	1.0 × 10^15^	1.0 × 10^15^

### 2.3. Model validation

To validate the reliability of our SCAPS-1D simulation framework, we compared the simulated output parameters of the unoptimized FASnI_3_-based PSC (with baseline parameters from Tables 1 and 2) with previously reported experimental and simulation results. As shown in Table 3, our simulated device yields a *J*_SC_ of ∼25.1 mA cm^−2^, a *V*_OC_ of ∼0.96 V, an FF of ∼80.2%, and a PCE of ∼19.3%. These values are in good agreement with the experimental work by Zhu *et al.*,^41^ who reported a *V*_OC_ of 0.911 V and a PCE of 14.06% or 13.73% for FASnI_3_-based PSCs with trimethylthiourea treatment,^62^ and with the simulation study by Muslimawati *et al.*,^47^ who obtained a PCE of ∼24.7% for FASnI_3_ under similar conditions. The slight differences can be attributed to variations in layer thicknesses, defect densities, and the absence of parasitic resistances in our ideal model. Furthermore, the relative performance ranking among the four absorber materials (FASnI_3_ > CsSnI_3_ > KGeCl_3_ > CsGeI_3_) is consistent with the trend reported by Shrivastav *et al.* for KGeCl_3_ and by other literature on CsGeI_3_.^44^ This validation confirms that our simulation parameters and modeling approach are reasonable and capable of producing physically meaningful comparative results.

**Table 3:** Different simulation parameters in the perovskite layers

Parameter	This work (simulated)	Zhu *et al.* (experimental)^41^	Muslimawati *et al.* (simulated)^47^
*V*_OC_ (V)	0.96	0.911	1.277
*J*_SC_ (mA cm^−2^)	25.1	0.82	22.4
FF (%)	80.2	75.69	86.36
PCE (%)	19.3	14.06	24.7

## 3. Results and discussion

### 3.1. Device performance of different absorber layers

Within perovskite solar cells (PSCs), perovskite materials serve as critical components for improving device efficiency. They enable effective charge migration and contribute to the photovoltaic process, yet internal defects may trigger performance degradation. Optimizing these material parameters significantly elevates cell stability and energy output, thereby playing a vital role in advancing device optimization.

**Fig. 2:**
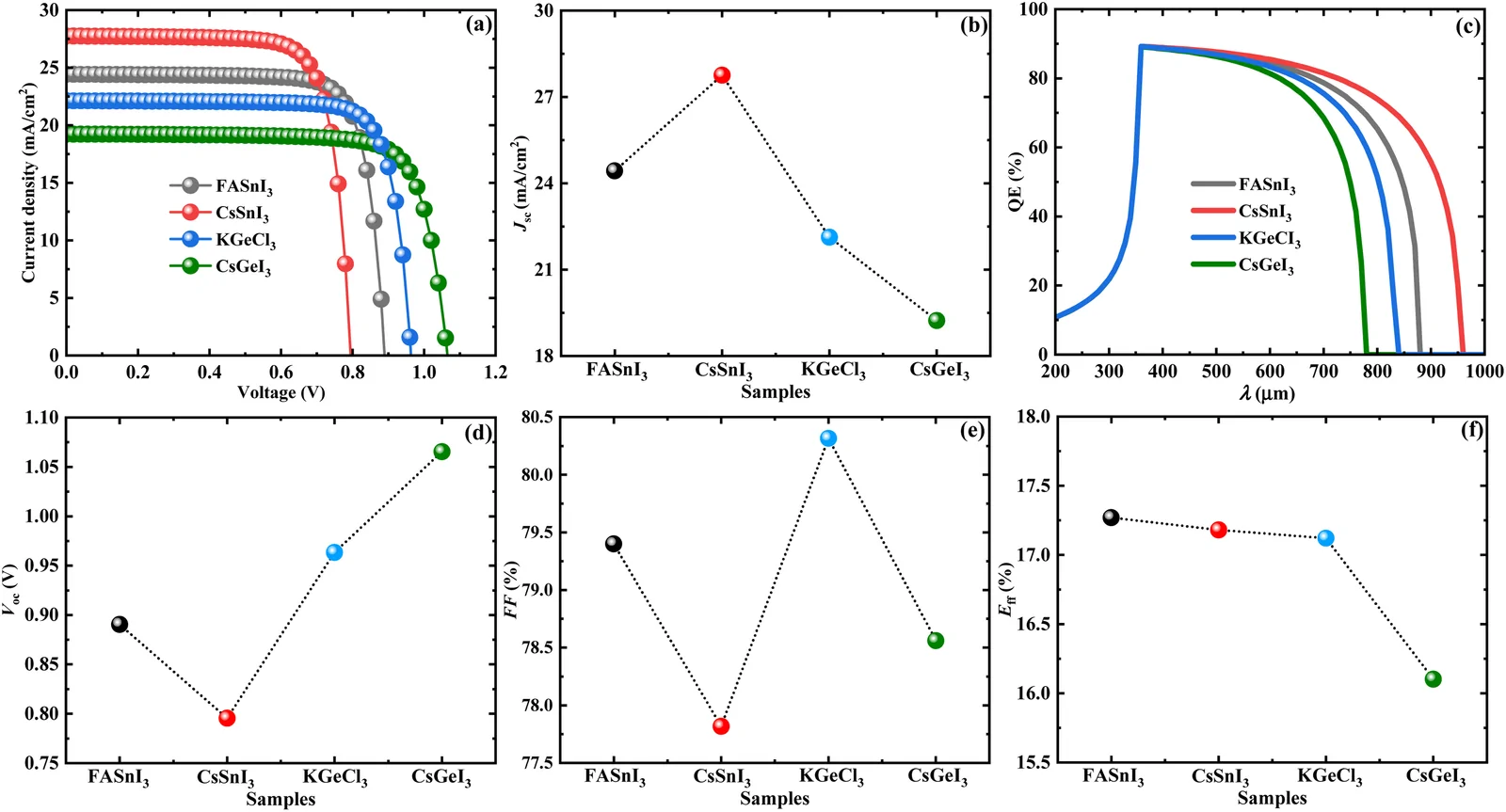
Comparative analysis of PSCs with distinct absorber layers. (a) Current–voltage (*J*–*V*) profiles, (b) short-circuit current density (*J*_SC_), (c) external quantum efficiency (EQE), (d) open-circuit voltage (*V*_OC_), (e) fill factor (FF), and (f) overall efficiency metrics of samples.

To investigate the impact of diverse perovskite materials on the performance of perovskite solar cells (PSCs), device models were constructed using SCAPS-1D software, incorporating active layers of FASnI_3_, CsSnI_3_, KGeCl_3_, and CsGeI_3_. The simulation results are illustrated in Fig. 2. Fig. 2(a) displays the current–voltage (*J*–*V*) characteristics of solar cells with these different perovskite materials. It is worth noting that there are significant differences in the short-circuit current density (*J*_SC_) and open-circuit voltage (*V*_OC_) of PSCs. For clearer comparison, the *J*_SC_ values are extracted and presented in Fig. 2(b). Among all samples, CsSnI_3_ exhibits the highest *J*_SC_ (∼27.8 mA cm^−2^), trailed by FASnI_3_ (∼24.4 mA cm^−2^). KGeCl_3_ and CsGeI_3_ show lower *J*_SC_ values compared to the other two. The progressive decline in *J*_SC_ along the sequence CsSnI_3_ → FASnI_3_ → KGeCl_3_ → CsGeI_3_ indicates that CsSnI_3_ and FASnI_3_ (the two absorbers with narrower bandgaps) exhibit superior charge absorption and/or reduced interface recombination compared to KGeCl_3_ andCsGeI_3_. Fig. 2(c) presents the external quantum efficiency (EQE) spectra of different solar cells as a function of wavelength. It can be observed that the EQE profiles at long-wavelength response of the solar cell decreases successively from CsSnI_3_, FASnI_3_, KGeCl_3_, to CsGeI_3_. This behavior is attributed to the stepwise increase in the absorption layer's bandgap. As the bandgap expands, the material's absorption efficiency for long-wavelength light diminishes because these wavelengths fall below the energy threshold required for electronic transitions. The resulting reduction in light absorption directly translates to lower EQE values, reflecting impaired charge carrier generation at extended wavelengths.

The decreasing trend of short-circuit current density (*J*_SC_) from CsSnI_3_ (≈27.8 mA cm^−2^) to CsGeI_3_ (≈15.7 mA cm^−2^), as shown in Fig. 2(b), is primarily governed by the increasing bandgap of the absorber layers (Table 2: CsSnI_3_ 1.3 eV, FASnI_3_ 1.4 eV, KGeCl_3_ 1.49 eV, CsGeI_3_ 1.6 eV). A wider bandgap reduces the absorption of long-wavelength photons, as directly evidenced by the lower external quantum efficiency (EQE) of CsGeI_3_ across the 500–800 nm range (Fig. 2(c)). Although germanium itself does not directly cause the lower *J*_SC_, the CsGeI_3_ compound exhibits a larger bandgap compared to tin-based analogues, which is an intrinsic property of germanium halide perovskites. This wider bandgap limits the solar spectrum utilization, resulting in fewer photogenerated carriers and thus a lower *J*_SC_. While CsGeI_3_ delivers the highest open-circuit voltage (*V*_OC_ ≈ 1.14 V, Fig. 2(d)) due to its larger bandgap, the overall power conversion efficiency (PCE) depends on the product of three parameters:4
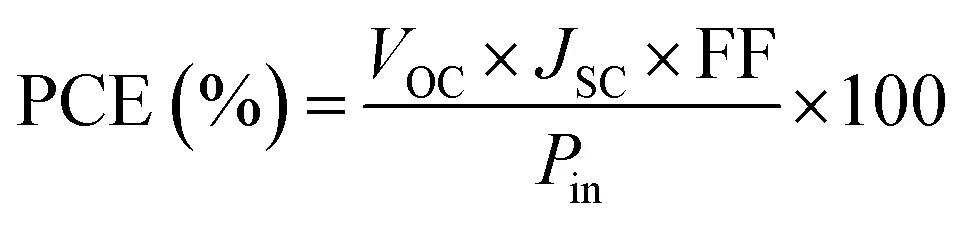
where *P*_in_ is the incident light power density (100 mW cm^−2^ under AM 1.5G). For CsGeI_3_, despite its high *V*_OC_ the significantly lower *J*_SC_ (≈15.7 mA cm^−2^) and moderate fill factor (FF ≈ 69.5%, Fig. 2(e)) cannot compensate for the current loss. In contrast, FASnI_3_ achieves a balanced combination: a moderate bandgap (1.4 eV) yields a reasonably high *J*_SC_ ≈ 24.4 mA cm^−2^ and a competitive *V*_OC_ (≈0.92 V), together with a good FF (≈74.6%), resulting in the highest PCE (≈16.7%). This trade-off is well described by the Shockley–Queisser limit, where an optimal bandgap around 1.3–1.4 eV maximizes PCE. Therefore, the lower performance of CsGeI_3_ is not due to germanium being inherently inferior, but rather because its wider bandgap (1.6 eV) is suboptimal for single-junction solar cells under the AM 1.5G spectrum. Future strategies such as alloying with tin or using tandem configurations could help overcome this limitation.

**Fig. 3:**
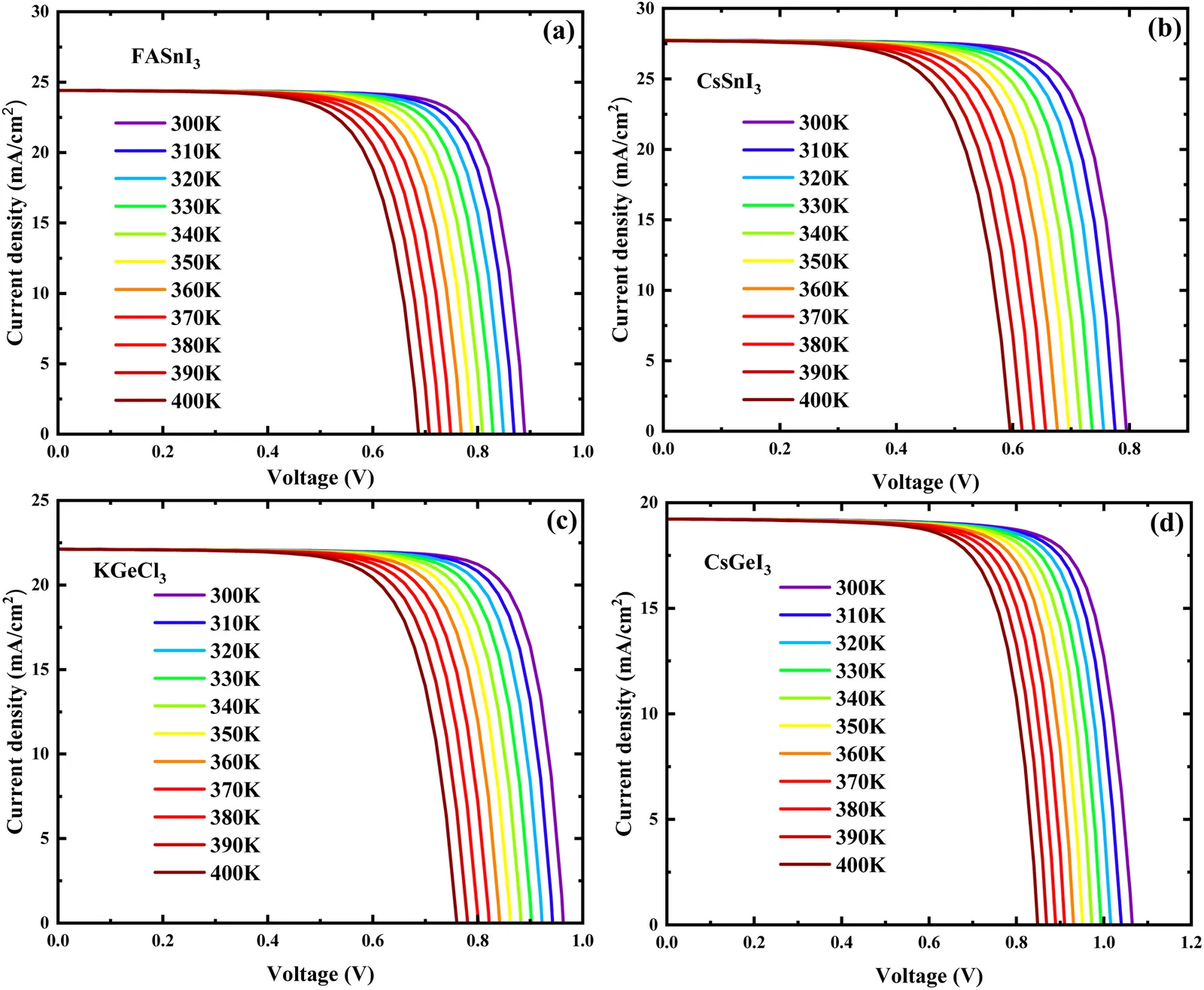
Temperature-dependent current–voltage characteristics of solar cells with different absorber layers through simulation.

The *V*_OC_ of different samples are displayed in Fig. 2(d). The *V*_OC_ values are observed to increase in the order of CsSnI_3_, FASnI_3_, KGeCl_3_, and CsGeI_3_. This phenomenon occurs because the band gap of the absorber layer gradually increases. As the potential difference from the separation of photogenerated carriers grows larger, *V*_OC_ rises accordingly. To further uncover the cause of *V*_OC_ changes in solar cells, temperature-dependent current–voltage measurement was carried out on devices with varying buffer layers. Solar cells typically function stably between 300 K and 400 K, but extreme climates, such as desert summers or polar winters, induce large temperature variations.^20,50^ Different photovoltaic devices show unique temperature response traits. Fig. 3 reveals that working point temperature shifts minimally affect *J*_SC_ but significantly impact *V*_OC_. *V*_OC_ consistently drops as operational temperature rises due to thermal activation of transient defects at high temperatures, which increase intrinsic defect densities in semiconductor materials. This leads to enhanced recombination and measurable *V*_OC_ reduction under high-temperature conditions. The interface recombination activation energy (*E*_a_) is a crucial parameter influencing the performance of thin-film solar cells, such as PSCs, CIGS, and CdTe, *etc.*^8,55,63^ The efficiency of solar cells is severely constrained at heterojunction interfaces due to recombination centers, typically induced by lattice mismatches and energy level disparities, as well as the extent of the charge depletion region. These factors promote non-radiative recombination of carriers. Accurately identifying these centers necessitates measuring the activation energy *E*_a_, which quantifies the energy barrier involved in recombination. The temperature dependence of *V*_OC_ plays a pivotal role in this method, and the underlying relationship is expressed through an equation:^8,23,64,65^5
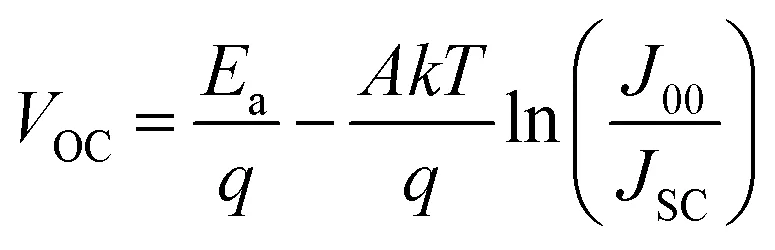


**Fig. 4:**
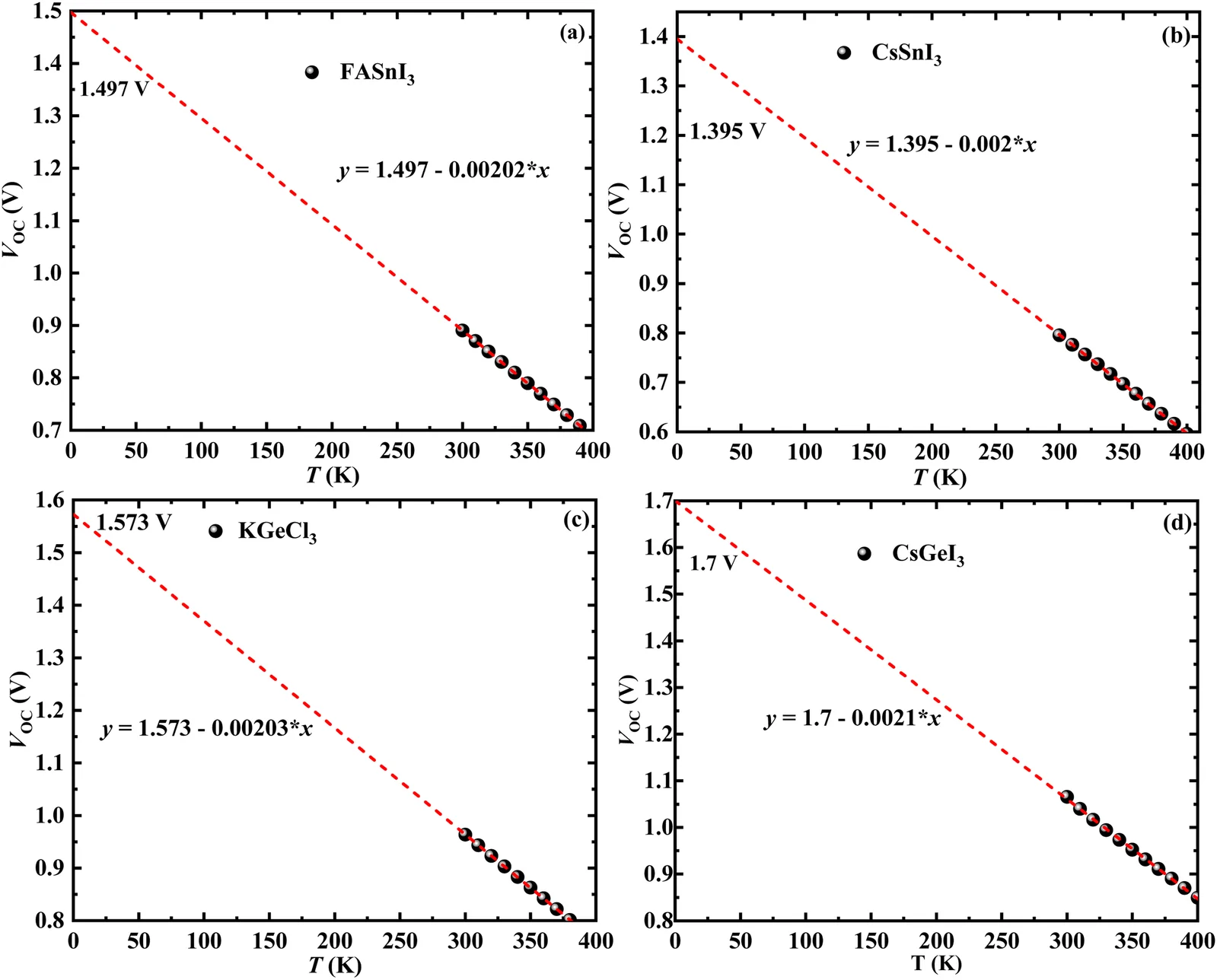
Activation energy of PSCs with the absorber layers of (a) FASnI_3_, (b) CsSnI_3_, (c) KGeCl_3_, and (d) CsGeI_3_, determined by temperature-dependent current–voltage analysis.

The variables *J*_00_, *T*, *A*, *q*, and *k* are defined as follows: *J*_00_ is the pre-factor influenced by temperature, *T* is the temperature, *A* is the diode quality factor, *q* is the electric charge quantity, and *k* is the Boltzmann constant. Upon deducing the temperature *T* to 0 K, the intercept values can be treated as the *E*_a_ value. The detailed methodology for calculating the *E*_a_ of PSCs is illustrated in Fig. 4. This analysis involves extrapolating the *V*_OC_ to the theoretical work temperature point of 0 K, a critical step for isolating the intrinsic electronic properties of the materials from thermal effects. The extrapolated *V*_OC_ values for the four investigated materials, CsSnI_3_, FASnI_3_, KGeCl_3_, and CsGeI_3_, are systematically determined as 1.497 V, 1.395 V, 1.573 V, and 1.700 V, respectively. These values are subsequently converted to *E*_a_, yielding 1.497 eV, 1.395 eV, 1.573 eV, and 1.700 eV for each material, respectively. This conversion leverages the fundamental relationship between *V*_OC_ and *E*_a_, where the latter represents the energy barrier for charge carrier recombination.

The observed higher *E*_a_ values for CsGeI_3_-based PSCs can be attributed to their superior energy level alignment at the absorber/buffer interface. This favorable alignment minimizes the formation of defect states, thereby significantly suppressing interface recombination losses. In contrast, the lower *E*_a_ values observed devices indicate a pronounced non-radiative recombination mechanism. This discrepancy stems from the migration of recombination centers from the bulk material region to the physical interface between the absorber and buffer layers. Such migration creates localized energy traps, which facilitate the capture and subsequent recombination of charge carriers. Consequently, charge carriers in PSCs are more prone to recombine at these interfaces.

The lower photovoltaic performance of CsGeI_3_ among the four lead-free absorbers can be attributed to several interrelated material properties. First, CsGeI_3_ has the widest bandgap (1.6 eV, Table 2) among the four materials. While a wider bandgap generally increases the open-circuit voltage (*V*_OC_) in principle, it severely limits light absorption in the long-wavelength region, as evidenced by its lowest EQE across the entire visible and near-infrared range (Fig. 2(c)). Consequently, CsGeI_3_ exhibits the lowest short-circuit current density (*J*_SC_ ≈ 15.7 mA cm^−2^, Fig. 2(b)) despite having a relatively high *V*_OC_. Second, the electron affinity (*χ* = 3.6 eV, Table 2) and dielectric constant (*ε*_r_ = 18) of CsGeI_3_ differ substantially from those of the TiO_2_ ETL and Spiro-OMeTAD HTL. This mismatch likely leads to suboptimal energy level alignment at both interfaces, increasing interface recombination losses. Third, although CsGeI_3_ has reasonable carrier mobilities (*µ*_n_ = *µ*_p_ = 20 cm^2^ V^−1^ s^−1^), its relatively low acceptor doping concentration (*N*_A_ = 1 × 10^15^ cm^−3^, Table 2) compared to FASnI_3_ (1 × 10^16^ cm^−3^) reduces the built-in electric field and hole extraction efficiency. Fourth, the higher dielectric constant (*ε*_r_ = 18) may enhance dielectric screening but can also promote polaron formation and trap-assisted recombination. Combined with a moderate defect density (1 × 10^15^ cm^−3^), these factors collectively result in the lowest fill factor (FF) and power conversion efficiency (PCE) among the four materials. Therefore, despite its lead-free and stable nature, CsGeI_3_ requires further compositional or interface engineering to overcome its bandgap-related absorption limitations and energy-level mismatches.

Fig. 2(e and f) illustrates the FF and PCE characteristics of PSCs with the absorber layers comprising CsSnI_3_, FASnI_3_, KGeCl_3_, and CsGeI_3_. In Fig. 2(e), KGeCl_3_ attains the peak FF, trailed by FASnI_3_, while CsGeI_3_ and CsSnI_3_ show minimal FF values. In contrast, Fig. 2(f) highlights FASnI_3_'s dominance in PCE, with CsSnI_3_ and KGeCl_3_ as the intermediate performer and CsGeI_3_ as the least efficient. Combining these trends with earlier data, it is evident that FASnI_3_ provide the highest comprehensive photovoltaic performance. Consequently, subsequent investigations are directed toward the FASnI_3_-based PSCs.

The superior PCE of FASnI_3_ over the other three lead-free perovskites can be attributed to a combination of its favourable optoelectronic properties. First, FASnI_3_ possesses a moderate bandgap of 1.4 eV (Table 2), which is close to the optimal value predicted by the Shockley–Queisser limit for single-junction solar cells. This bandgap enables a balanced trade-off between light absorption and open-circuit voltage: a narrower bandgap (*e.g.*, 1.3 eV for CsSnI_3_) enhances short-circuit current density (*J*_SC_) but reduces *V*_OC_ due to higher intrinsic carrier recombination, while a wider bandgap (1.49 eV for KGeCl_3_ and 1.6 eV for CsGeI_3_) limits photon harvesting in the long-wavelength region, as evidenced by their lower EQE in Fig. 2(c). Second, the electron affinity (*χ* = 3.3 eV) and dielectric constant (*ε*_r_ = 8.2) of FASnI_3_ provide suitable energy level alignment with both the TiO_2_ electron transport layer (ETL) and the Spiro-OMeTAD hole transport layer (HTL), facilitating efficient charge extraction and reducing interface recombination. Third, although CsSnI_3_ exhibits higher electron mobility (*µ*_n_ = 1500 cm^2^ V^−1^ s^−1^. 22 cm^2^ V^−1^ s^−1^ for FASnI_3_, Table 2), its PCE is lower mainly because of a much higher defect tolerance. In fact, the simulated defect densities are identical (1 × 10^15^ cm^−3^) for all absorbers, but the combination of a smaller bandgap and possibly higher non-radiative recombination at interfaces leads to a pronounced *V*_OC_ deficit (Fig. 2(d)). Finally, the balanced electron and hole mobilities of FASnI_3_ (both 22 cm^2^ V^−1^ s^−1^) support efficient bipolar transport, whereas the large mobility disparity in CsSnI_3_ (*µ*_n_ = 1500, *µ*_p_ = 25) may cause space-charge accumulation and enhance recombination. These combined factors – optimal bandgap, suitable energy alignment, balanced carrier transport, and reasonable defect tolerance – make FASnI_3_ the most comprehensively efficient lead-free absorber among the four materials under the simulated conditions.

### 3.2. Device performance of FASnI_3_-based PSCs

In FASnI_3_-based PSCs, both the thickness and carrier concentration of the electron transport layer (ETL) are critical factors influencing device performance.^66,67^ The thickness determines the carrier transport distance and recombination likelihood. Excessive thickness extends the transport path, increases recombination, and reduces efficiency, while insufficient thickness may compromise electron transport stability and integrity. Optimal thickness facilitates efficient electron transport to the electrode, minimizes recombination losses, and enhances cell efficiency. Carrier concentration also plays a vital role in electron transport capacity and charge balance. Low concentration limits electron supply and current output, whereas high concentration may cause charge buildup and increased recombination. Appropriate carrier concentration ensures smooth electron transport, maintains internal charge balance, and optimizes performance.

**Fig. 5:**
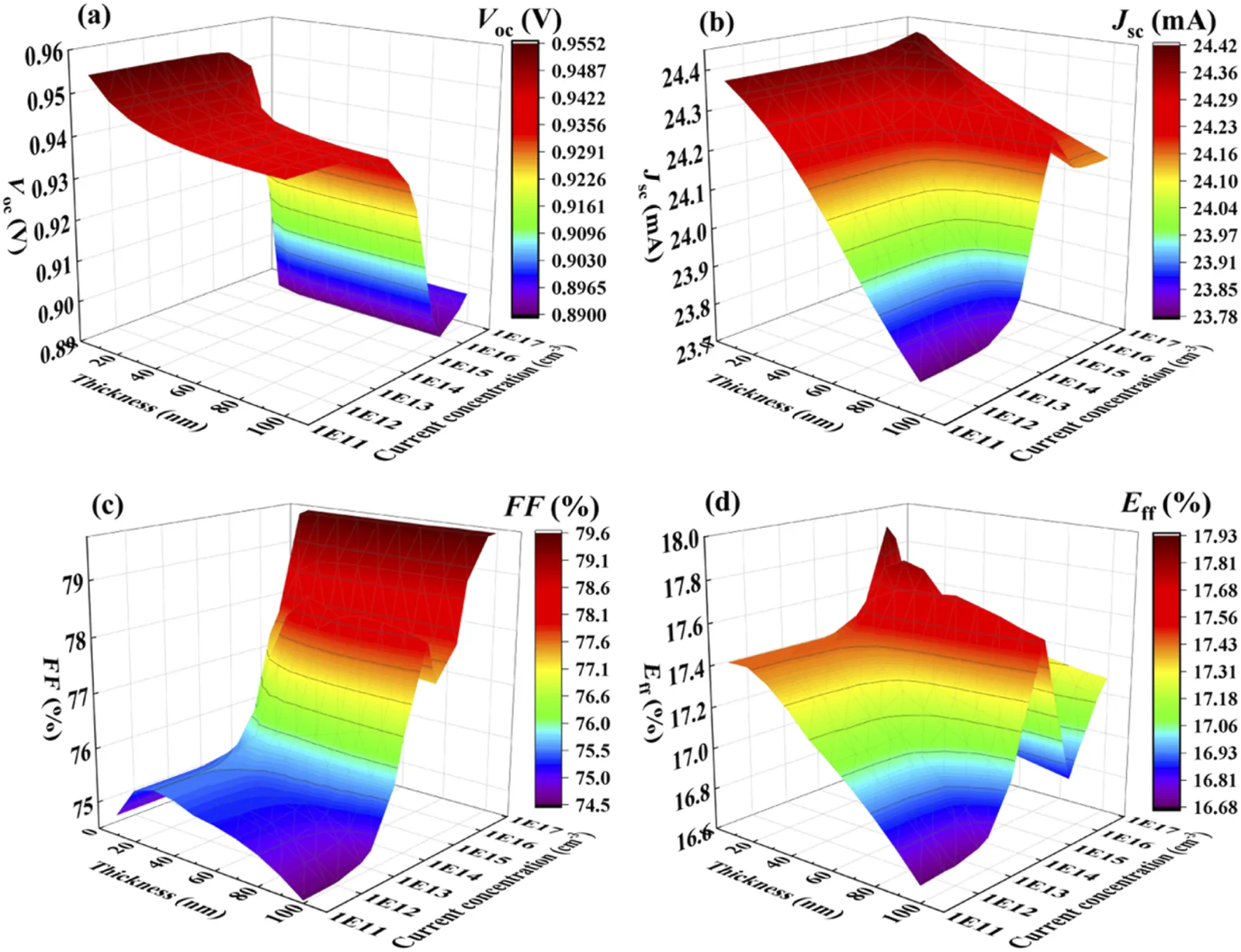
3D surface plot showing the simultaneous effects of TiO_2_ layer thickness and carrier concentration on solar cell parameters: (a) *V*_OC_, (b) *J*_SC_, (c) FF, (d) *E*_ff_.

**Fig. 6:**
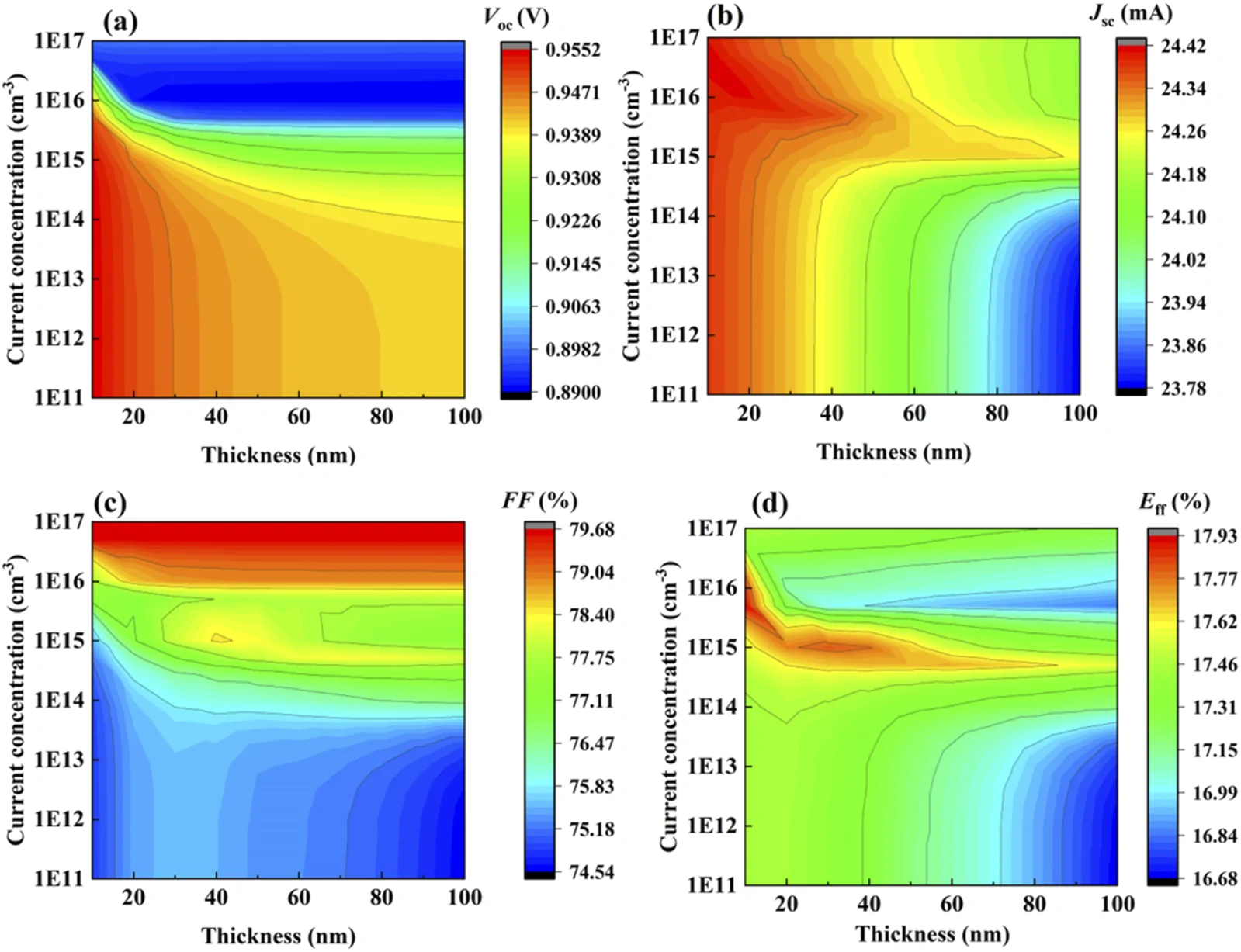
Contour plots demonstrating the simultaneous impact of thickness and carrier concentration variations on photovoltaic performance: (a) *V*_OC_, (b) *J*_SC_, (c) FF, (d) *E*_ff_.

To further explore the impact of TiO_2_ ETL on PSCs performance, a systematic study was conducted by adjusting ETL thickness and carrier concentration. Simulations varied ETL thickness from 0.1 µm to 1 µm in 0.1 µm increments, with carrier concentrations ranging from 1 × 10^11^ to 1 × 10^17^ cm^−3^. The 3D surface plot in Fig. 5 illustrates simulation results, and contour plots in Fig. 6 provide a clearer quantitative view. Below we provide a detailed physical interpretation of the observed trends.

For clearer comparison, photovoltaic (PV) parameters, *J*_SC_, *V*_OC_, FF, and PCE, were extracted and presented in Fig. 6. As observed in Fig. 6(a), the open-circuit voltage initially decreases and then stabilizes with increasing ETL thickness. This behavior arises because a thicker ETL increases the series resistance and the distance that photogenerated electrons must travel, enhancing the probability of bulk recombination within the TiO_2_ layer before electrons reach the FTO contact. When the ETL is very thin (≈0.1 µm), recombination losses are minimized, yielding a higher *V*_OC_. As thickness increases beyond ≈0.4 µm, the additional recombination saturates because the electron flux becomes limited by diffusion rather than further thickness increases. Regarding carrier concentration: at low doping levels (<10^13^ cm^−3^), the ETL has low conductivity, and the built-in field extends deeper into the perovskite, which helps maintain a high *V*_OC_. Increasing the carrier concentration raises the Fermi level in the ETL, reducing the band bending at the ETL/perovskite interface and thereby slightly lowering *V*_OC_. Beyond a critical concentration (∼10^15^ cm^−3^), the ETL becomes degenerately doped, and further increases have negligible effect on the electrostatic potential distribution.

From Fig. 6(b), it can be seen that the *J*_SC_ decreases monotonically with ETL thickness because a thicker layer introduces more optical absorption (despite TiO_2_ being transparent, some parasitic absorption remains) and increases series resistance, both of which reduce the collected current. At low thicknesses (0.1–0.3 µm), a higher carrier concentration increases *J*_SC_ because the enhanced conductivity reduces ohmic losses and improves electron collection. However, at large thicknesses (>0.6 µm), the resistive loss dominates regardless of doping, and carrier concentration no longer influences *J*_SC_ significantly. This explains why the contour lines become vertical (thickness-limited) in the thick-ETL region.

As shown in Fig. 6(c), the fill factor shows a non-monotonic trend with ETL thickness at low carrier concentrations: it first decreases, then increases. The initial decrease (from 0.1 to 0.3 µm) is due to increased series resistance from the thicker ETL, which reduces the slope of the *J*–*V* curve near open circuit. The subsequent increase (beyond 0.4 µm) occurs because a moderately thick ETL provides better coverage, reducing pinhole-induced shunt paths and improving the rectifying behavior of the junction. At high carrier concentrations (>10^15^ cm^−3^), the ETL is sufficiently conductive that series resistance is negligible, so thickness has little impact on FF, and FF remains high (≈78–80%). Furthermore, at any fixed thickness, a higher carrier concentration increases FF by reducing the ETL's series resistance, which steepens the *J*–*V* curve near the maximum power point.

The power conversion efficiency combines the above trends. At low carrier concentrations, PCE initially rises with ETL thickness due to improved junction quality (reduced shunting) up to an optimum (∼0.4–0.6 µm), then slightly declines because series resistance eventually overcomes the shunting benefit (Fig. 6(d)). At high carrier concentrations, the PCE is high and stable over a wide thickness range (0.3–0.8 µm) because the low resistive losses mask thickness-induced recombination. The optimum PCE is achieved with an ETL thickness of approximately 0.5 µm and a carrier concentration of around 10^16^ cm^−3^, balancing efficient charge extraction, minimal recombination, and low series resistance.

**Fig. 7:**
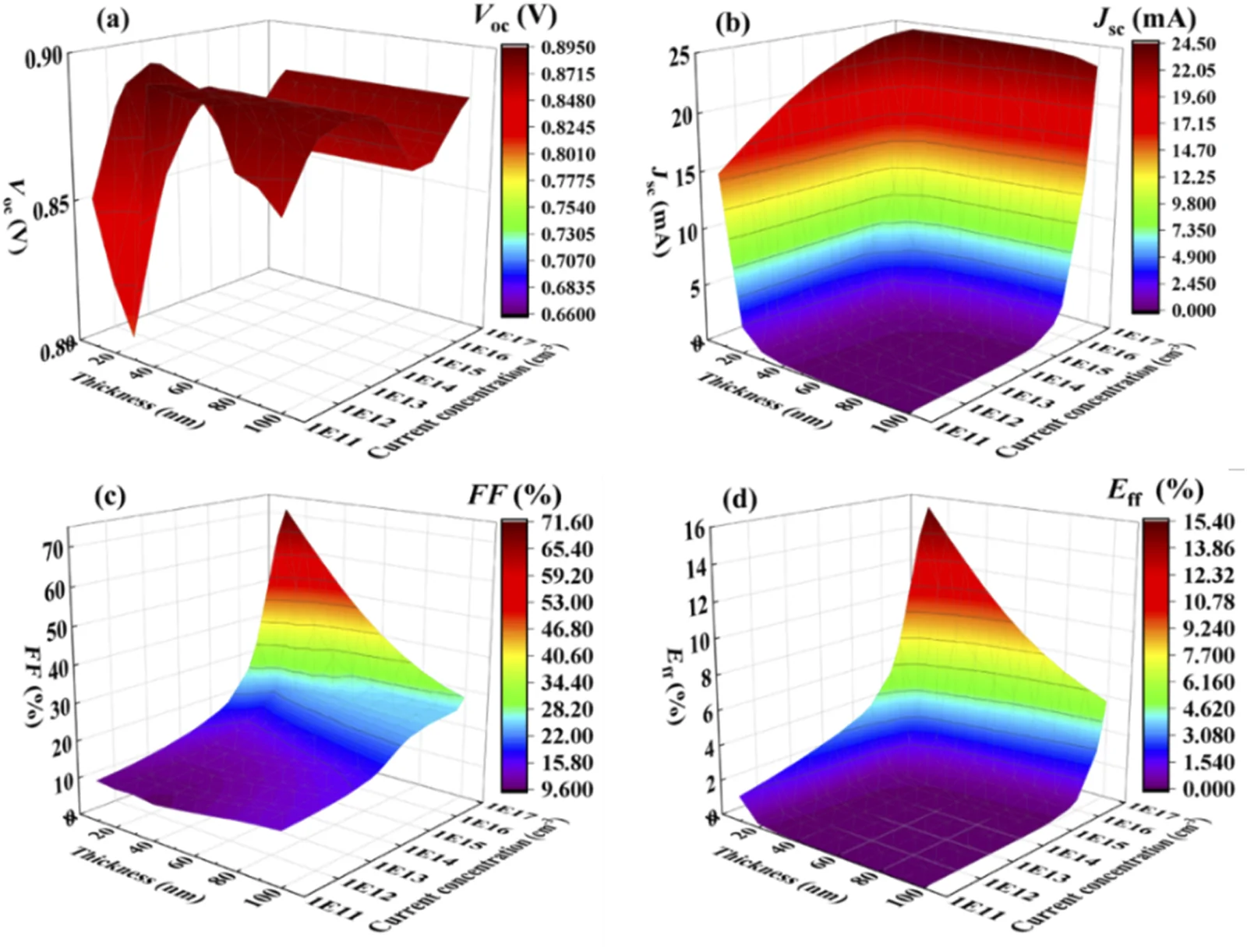
3D surface plot showing the simultaneous effects of Spiro-OMeTAD HTL layer thickness and carrier concentration on solar cell parameters: (a) *V*_OC_, (b) *J*_SC_, (c) FF, (d) *E*_ff_.

**Fig. 8:**
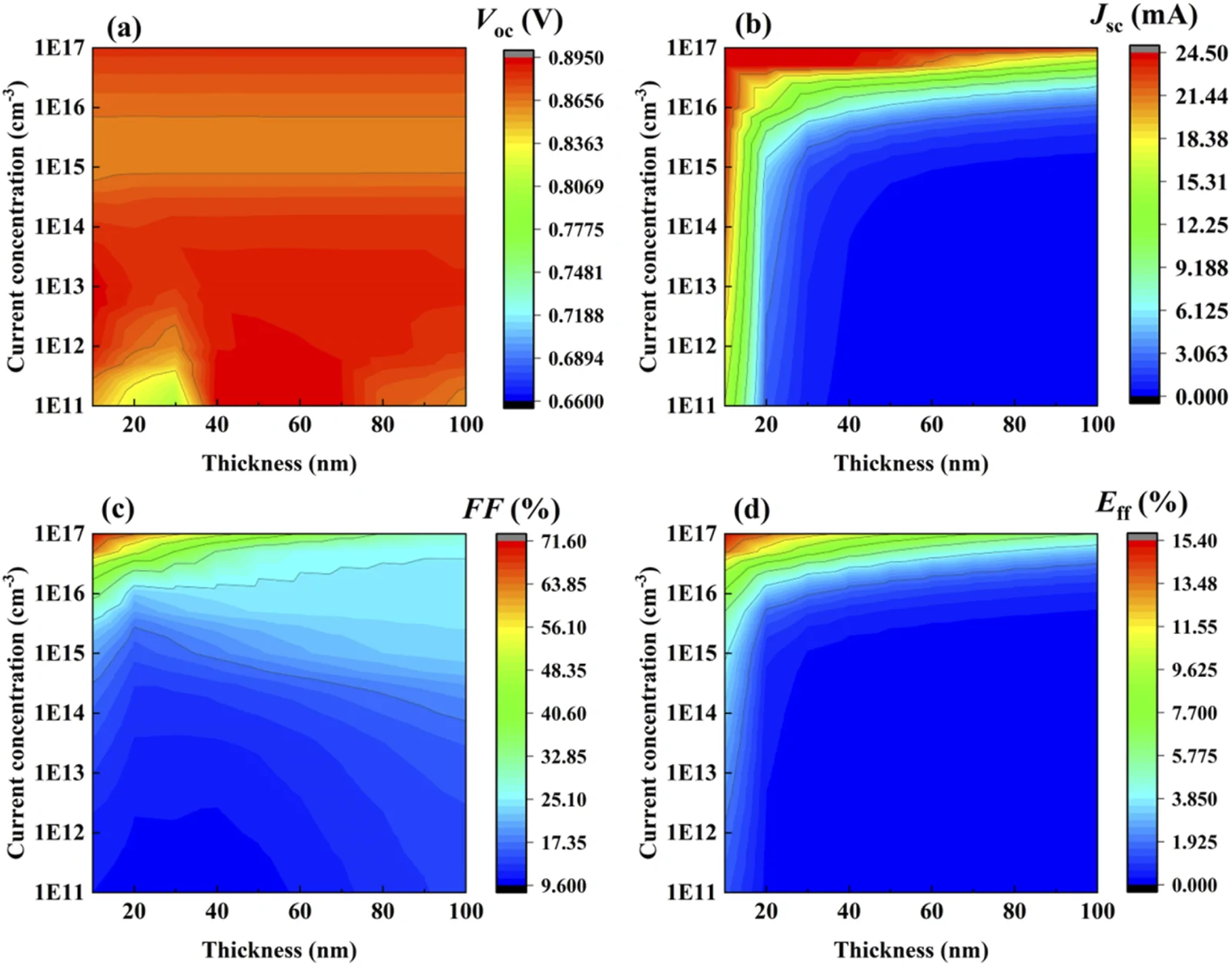
Contour plots demonstrating the simultaneous impact of HTL thickness and carrier concentration variations on photovoltaic performance: (a) *V*_OC_, (b) *J*_SC_, (c) FF, (d) *E*_ff_.

The thickness and carrier concentration of the HTL (Hole Transport Layer) significantly influence the performance of PSCs. An optimal thickness enhances hole extraction and transport while minimizing recombination losses; excessive thickness, however, can impede carrier mobility. Similarly, an appropriate carrier concentration ensures efficient charge transfer; too low may hinder conductivity, while too high could lead to energy level misalignment. Balancing HTL thickness and carrier concentration is crucial for maximizing PSC efficiency and stability. For FASnI_3_-based PSCs, its results are exhibited in Fig. 7. It can be observed that the thickness and carrier concentration have a significant impact on the output parameters of the solar cell. In order to get a clearer comparison, the contour plots of FASnI_3_-based PSCs were displayed in Fig. 8.

The performance of perovskite solar cells (PSCs) is closely related to the thickness of the hole-transport layer (HTL) and the carrier concentration within it. In terms of *V*_OC_, at low carrier concentrations, *V*_OC_ may first decrease and then stabilize or increase slightly with increasing HTL thickness due to the interplay of factors such as charge accumulation and series resistance. At high carrier concentrations, the influence of HTL thickness on *V*_OC_ is less significant, and it remains at a relatively high level. For *J*_SC_, at low carrier concentrations, an increase in HTL thickness generally leads to a decrease in *J*_SC_ due to increased resistance for hole transport. However, at high carrier concentrations, the negative impact of thickness on *J*_SC_ is mitigated, and it can remain stable or even improve within a certain thickness range. Regarding the FF, at low carrier concentrations, FF is highly sensitive to HTL thickness, and an optimal thickness exists for high FF. At high carrier concentrations, the dependence of FF on HTL thickness is reduced, and FF can maintain a relatively high value over a broader thickness range. The PCE contour plot shows that there is an optimal combination of HTL thickness and carrier concentration to achieve the highest PCE. At low carrier concentrations, the PCE is generally low and greatly affected by HTL thickness, while at high carrier concentrations, the PCE can be significantly improved, and the optimal thickness range for high PCE expands. In conclusion, both HTL thickness and carrier concentration play crucial roles in determining the performance of PSCs, and optimizing these two factors is essential for achieving high-efficiency PSCs.

**Fig. 9:**
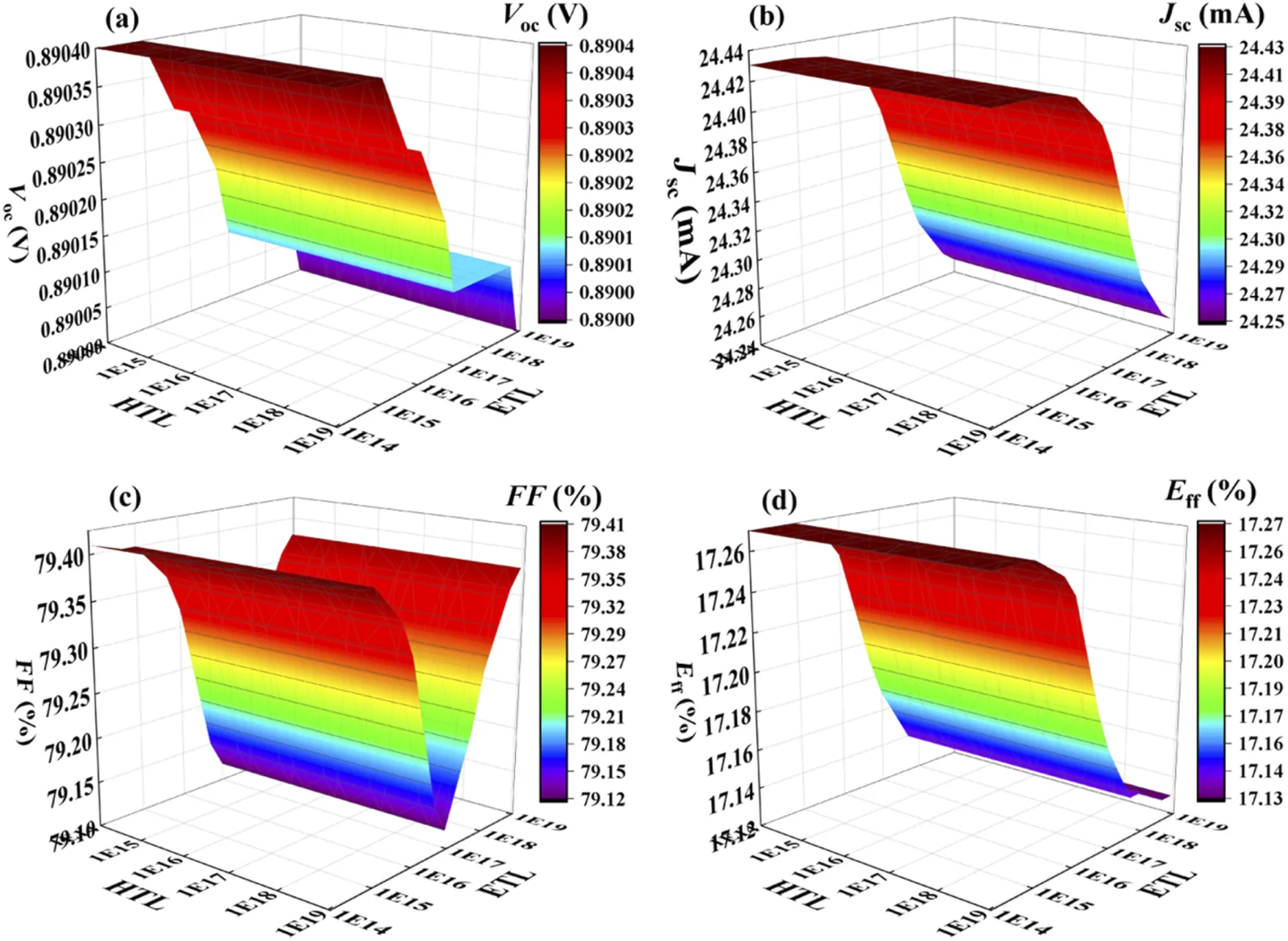
3D surface plot showing the simultaneous effects of defect concentration of ETL and HTL on solar cell parameters: (a) *V*_OC_, (b) *J*_SC_, (c) FF, (d) *E*_ff_.

The defect density of the ETL and HTL significantly impacts the performance of PSCs. An increase in the defect density of the ETL makes electrons more prone to recombination during the transport process, leading to a decrease in electron transport efficiency and, consequently, a reduction in the short-circuit current of the cell. Simultaneously, defects can act as carrier recombination centers, reducing carrier lifetime and affecting the fill factor and open-circuit voltage of the cell. An elevation in the defect density of the HTL impedes the effective transport of holes, increases the difficulty of hole extraction, and reduces hole collection efficiency, similarly exerting a negative influence on the short-circuit current. Moreover, defects exacerbate carrier recombination, causing a decline in the open-circuit voltage and fill factor of the cell. Therefore, reducing the defect density of both the ETL and HTL is of paramount importance for enhancing the electron and hole transport efficiency in PSCs, minimizing carrier recombination, and improving various performance indicators of the cell. In order to verify the influence laws of defect density in the ETL and HTL layers on the working mechanism of solar cells, the impact of different defect densities on the performance of solar cells was simulated, and the results are shown in Fig. 9.

**Fig. 10:**
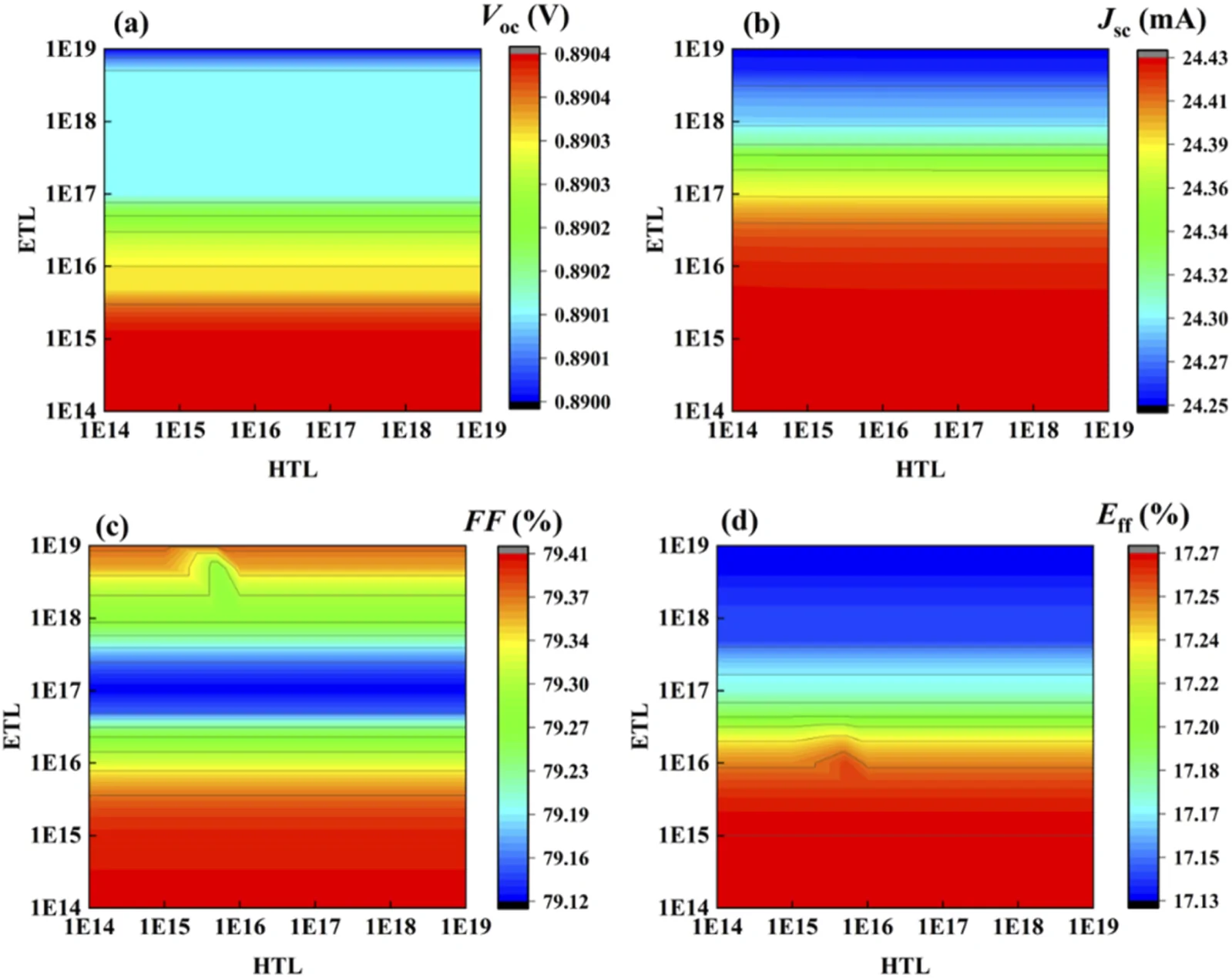
Contour plots demonstrating the simultaneous impact of defect densities variations on photovoltaic performance: (a) *V*_OC_, (b) *J*_SC_, (c) FF, (d) *E*_ff_.

To optimize the observational outcome, the 3D parameter visualizations presented in Fig. 10 are converted into contour mappings that depict variations with defect densities of ETL and HTL. The defect densities of ETL and HTL have multifaceted impacts on the performance of PSCs. As the ETL defect density increases, the *V*_OC_ shows a downward trend because these defects enhance the probability of electron–hole recombination, reducing the number of effective carriers reaching the electrodes. In contrast, changes in HTL defect density have a relatively minor effect on *V*_OC_, which remains stable within a low range of HTL defect densities. An increase in ETL defect density also negatively affects the *J*_SC_ by impeding electron transport and collection. While higher HTL defect density similarly reduces *J*_SC_, its impact is less pronounced than that of ETL. The influence of ETL defect density on the fill factor (FF) exhibits a distinct threshold behavior. FF remains relatively high and stable at low defect densities (<10^14^ cm^−3^) but decreases sharply once the defect density exceeds approximately 10^15^ cm^−3^. This decline is attributed to enhanced Shockley–Read–Hall (SRH) recombination, reduced electron diffusion length, and partial screening of the built-in field. In contrast, the HTL defect density causes a more gradual decline in FF (Fig. 10(c)) because hole mobility in Spiro-OMeTAD is already extremely low, so the transport is less sensitive to additional trap states. This mechanistic understanding highlights the importance of keeping ETL defect densities below 10^14^ cm^−3^ to achieve high fill factors in lead-free PSCs. Both ETL and HTL defect density increments cause a reduction in power conversion efficiency. Resulting from the combined decrease in *V*_OC_, *J*_SC_, and FF. Notably, ETL defect density has a more significant impact on PCE, indicating that ETL quality plays a crucial role in PSCs performance.

The greater sensitivity of PCE to ETL defect density compared to HTL defect density can be explained by several interrelated factors rooted in semiconductor device physics. First, in the typical n-i-p configuration (FTO/TiO_2_/perovskite/Spiro-OMeTAD), the ETL (TiO_2_) is responsible for extracting and transporting photogenerated electrons from the perovskite absorber to the front electrode. Any increase in ETL defect density introduces additional trap states near the conduction band edge, which act as effective recombination centers for electrons. Because electrons have reasonably high mobility in TiO_2_ (20 cm^2^ V^−1^ s^−1^), they can efficiently reach defect sites, making recombination highly sensitive to ETL defect density. In contrast, the HTL (Spiro-OMeTAD) is an organic semiconductor with extremely low hole mobility (≈2.1 × 10^−3^ cm^2^ V^−1^ s^−1^). In this mobility-limited regime, adding more defect states does not proportionally increase recombination because the hole flux is already constrained by slow drift/diffusion. Second, energy level alignment plays a role: at the ETL/perovskite interface, defects can create mid-gap states that promote Shockley–Read–Hall (SRH) recombination, directly reducing electron collection efficiency, *J*_SC_, and FF. At the HTL side, the valence band offset (≈0.2 eV) creates a small energy barrier that reflects some holes back into the perovskite, reducing the number of holes available to recombine at HTL defects. Third, the built-in electric field is primarily sustained by the ETL/perovskite junction; defects in the ETL can partially screen this field, reducing charge separation and further increasing recombination. Finally, the ETL/perovskite interface is located at the illuminated front side where the photogeneration rate is highest, while the HTL/perovskite interface is at the rear side where the generation rate is lower. Consequently, defects at the front interface capture a larger absolute number of carriers. For these reasons, ETL optimization yields substantially larger performance gains than HTL optimization.

The smaller impact of HTL defect density on device performance (particularly on *V*_OC_) follows directly from the asymmetries described above. In the n-type FASnI_3_ absorber under illumination, electrons are majority carriers, and *V*_OC_ is primarily determined by the electron quasi-Fermi level at the ETL/perovskite interface. Defects in the HTL mainly affect hole extraction, but holes are minority carriers and their recombination has a smaller effect on the overall electrostatic potential difference. Additionally, the low hole mobility of Spiro-OMeTAD already limits hole transport, so additional defect states cause only a gradual decline in performance. The small valence band offset also reflects some holes back into the perovskite, further reducing the impact of HTL defects. Thus, while HTL quality should not be neglected, it is less critical than ETL quality.

To quantitatively assess the impact of ETL optimization, we compare the baseline device (using parameters from Tables 1 and 2 without ETL tuning) with an optimized device (ETL thickness ≈ 0.5 µm, carrier concentration ≈ 1 × 10^16^ cm^−3^, and ETL defect density reduced from 1 × 10^15^ cm^−3^ to 1 × 10^13^ cm^−3^). Table 4 summarizes the key parameters and performance metrics. The optimized device achieves a PCE improvement of 2.6 percentage points (from 16.7% to 19.3%), demonstrating that careful ETL engineering, particularly reducing defect density, is essential for high-efficiency lead-free PSCs. This insight reinforces our conclusion that experimental efforts should prioritize high-quality, low-defect ETL deposition and interface passivation.

**Table 4:** Comparison of baseline and optimized ETL parameters and corresponding PSC performance

Parameter	Baseline	Optimized	Change
ETL thickness (µm)	0.05	0.5	+0.45
ETL carrier concentration (cm^−3^)	9 × 10^16^	1 × 10^16^	↓
ETL defect density (cm^−3^)	1 × 10^15^	1 × 10^13^	↓
*V*_OC_ (V)	0.92	0.96	+0.04
*J*_SC_ (mA cm^−2^)	24.4	25.1	+0.7
FF (%)	74.6	80.2	+5.6
PCE (%)	16.7	19.3	+2.6

## 4. Conclusion

This work provides a comprehensive comparative and optimization study of lead-free perovskite solar cells. Simulations show that among FASnI_3_, CsSnI_3_, KGeCl_3_, and CsGeI_3_, FASnI_3_ delivers the highest baseline PCE (≈16.7%), followed by CsSnI_3_ (≈16.0%), KGeCl_3_ (≈13.9%), and CsGeI_3_ (≈11.4%). The superior performance of FASnI_3_ arises from its near-optimal bandgap (1.4 eV) and balanced carrier mobilities, while the wider bandgap (1.6 eV) of CsGeI_3_ limits *J*_SC_ despite a higher *V*_OC_—a direct Shockley–Queisser trade-off. Optimization of FASnI_3_-based devices reveals that ETL (TiO_2_) thickness (≈0.5 µm) and carrier concentration (≈10^16^ cm^−3^) maximize PCE to ≈19.3% when combined with low defect density. Quantitatively, reducing ETL defect density from 1 × 10^15^ cm^−3^ to 1 × 10^13^ cm^−3^ improves PCE by +2.6 percentage points, whereas the same reduction in HTL (Spiro-OMeTAD) defect density yields only ≈0.5% gain. This asymmetry is explained by the n-i-p configuration (front-illuminated ETL collects majority electrons), the high electron mobility of TiO_2_*vs.* extremely low hole mobility of the organic HTL, and the ETL/perovskite junction sustaining the built-in field. Based on these quantitative and physical insights, the following clear roadmap is proposed for experimental fabrication: (1) select FASnI_3_ as the absorber; (2) fabricate TiO_2_ ETL with thickness ≈500 nm, carrier concentration ≈10^16^ cm^−3^, and defect density <10^14^ cm^−3^; (3) prioritize ETL interface passivation to suppress non-radiative recombination; (4) consider inorganic HTLs for improved hole transport. Additionally, KGeI_3_, with its expected narrower bandgap (≈1.3–1.4 eV), warrants future experimental and simulation studies, as it may achieve PCE comparable to FASnI_3_. These findings offer a physically grounded direction for developing efficient and sustainable lead-free perovskite solar cells.

## Author contributions

Zhongjie Wang: writing – original draft, investigation, supervision, methodology, conceptualization, funding acquisition. Yuhao Wang: methodology, supervision, data curation, software. Benxiong Hu: investigation, conceptualization, data curation, validation. Chenxi Zheng: supervision, investigation, conceptualization, formal analysis. Jian Qu: data curation, visualization, methodology. Yunxiang Zhang: writing – review and editing, supervision, methodology, resources. Qinfang Zhang: conceptualization, investigation, resources, writing – review and editing.

## Conflicts of interest

The authors declare that they have no known competing financial interests or personal relationships that could have appeared to influence the work reported in this paper.

## Data Availability

The data that support the findings of this study are available from the corresponding author upon reasonable request.

## References

[cit1] Izam N. S. M. N., Itam Z., Sing W. L., Syamsir A. (2022). Energies.

[cit2] Zhang Y., Lin S., Cheng S., He Z., Hu Z., Zhou Z., Liu W., Sun Y. (2021). Engineering.

[cit3] Lin S., Liu W., Zhang Y., Cheng S., Fan Y., Zhou Z., He Q., Zhang Y., Sun Y. (2018). Sol. Energy Mater. Sol. Cells.

[cit4] Sun Y., Lin S., Li W., Cheng S., Zhang Y., Liu Y., Liu W. (2017). Engineering.

[cit5] Pei F., Lin S., Zhang Z., Lin S., Huang X., Zhao M., Xu J., Zhuang X., Zhang Y., Tang J., Chen Y., Li K., Wang L., Liu G., Qian D., Liu H., Zhou W., Chen Y., Wang J., Zhou H., Li B., Zhong D., Jiang Y., Chen Q. (2025). Nat. Energy.

[cit6] Keller J., Kiselman K., Donzel-Gargand O., Martin N. M., Babucci M., Lundberg O., Wallin E., Stolt L., Edoff M. (2024). Nat. Energy.

[cit7] Sadia H., Hasan S. A. U., Rehan S., Ali I., Siddiqui L., Abbas R., Siddique Y., Kwak S. K. (2026). Nano Energy.

[cit8] Zhang Y., Gao Q., Ao J., Zhang Y., Bi J., Guo J., Han Y., Sun G., Zhang Y., Liu W., Liu F. (2021). ACS Appl. Energy Mater..

[cit9] Chen Z., Huang L., Zhou X., Zhang Y., Chen X., Zhou C., Abdelsalam H., Chen W., Wang Z., Zhang Q. (2025). Environ. Res..

[cit10] Zhang Y., Wang H., Huang L., Chen J., Wu C., Nie K., Liang Y., Wang Z., Zhang Q. (2026). Mater. Lett..

[cit11] Chen W., Liang Y., Chen Z., Zhang Y., Wang J., Chen X., Zhou C., Nie K., Wang Z., Zhang Q. (2025). J. Alloys Compd..

[cit12] Zhang Y., Mu Z., Zhou C., Zhang Z., Chen Z., Cheng X., Abdelsalam H., Chen W., Khalafallah D., Zhang Q. (2024). Mater. Today Energy.

[cit13] Hu J., Li J., Liu X., Xiao W., Yu H., Abdelsalam H., Liu C., Zou Z., Zhang Q. (2025). J. Colloid Interface Sci..

[cit14] Tsai T.-Y., Zheng J.-R., Yuan C.-S., Chen T.-Y., Shen H. (2024). J. Environ. Chem. Eng..

[cit15] Zhang Y., Mu Z., Zhou C., Abdelsalam H., Zhou X., Shi L., Chen Z., Liang Y., Wang Z., Zhang Q. (2025). J. Mater. Chem. A.

[cit16] Zhang Y., Wang Y., Zhou C., Abdelsalam H., Chen W., Huang L., Mu Z., Chen Z., Ahmed D. H., Zhang Q. (2024). J. Mater. Sci. Technol..

[cit17] Zhang Y., Xu S., Chen J., Zhang J., Mu Z., Zhou C., Abdelsalam H., Zhang Q. (2026). Mater. Horiz..

[cit18] Dastgeer G., Nisar S., Zulfiqar M. W., Eom J., Imran M., Akbar K. (2024). Nano Energy.

[cit19] Gu Y., Zhou C., Chen W., Zhang Y., Yao Y., Zhou Z., Sun Y., Liu W. (2024). Appl. Phys. A.

[cit20] Qing G., Ao J., Zhang Y., Zhang Y., Guo J., Sun G., Liu W., Liu F., Zhang Y. (2021). ACS Appl. Energy Mater..

[cit21] Li Y., Wang Y., Xu Z., Peng B., Tran N. Q., Saxena K. K., Vadivel S., Liu X. (2025). J. Mater. Sci. Technol..

[cit22] Chirila A., Reinhard P., Pianezzi F., Bloesch P., Uhl A. R., Fella C., Kranz L., Keller D., Gretener C., Hagendorfer H., Jaeger D., Erni R., Nishiwaki S., Buecheler S., Tiwari A. N. (2013). Nat. Mater..

[cit23] Zhang Y., Hu Z., Lin S., Wang C., Cheng S., He Z., Zhou Z., Sun Y., Liu W. (2020). Sol. RRL.

[cit24] Li Q., Zheng Y., Wang H., Liu X., Lin M., Sui X., Leng X., Liu D., Wei Z., Song M., Li D., Yang H. G., Yang S., Hou Y. (2025). Science.

[cit25] Zai H., Yang P., Su J., Yin R., Fan R., Wu Y., Zhu X., Ma Y., Zhou T., Zhou W., Zhang Y., Huang Z., Jiang Y., Li N., Bai Y., Zhu C., Huang Z., Chang J., Chen Q., Zhang Y., Zhou H. (2025). Science.

[cit26] Li W., Xu Z., Yan Y., Zhou J., Huang Q., Xu S., Zhang X., Zhao Y., Hou G. (2024). Adv. Energy Mater..

[cit27] Zhou C., Chen W., Chen Z., Cheng X., Zhang Y., Sun G., Li K., Liu Z., Shi L., Wang Z., Liu W., Zhang Q. (2026). J. Opt..

[cit28] Zhang Y., Zhang Y., Chen X., Wang S., Gao Q., Wu M., Wang Z., Ao J., Sun Y., Liu W., Zhang Q. (2022). Mater. Sci. Semicond. Process..

[cit29] Walelgn A. Y., Geffe C. A., Aberra N., Zhang Y., Zhang Q. (2026). Mater. Sci. Eng., B.

[cit30] Kojima A., Teshima K., Shirai Y., Miyasaka T. (2009). J. Am. Chem. Soc..

[cit31] Xiong Z., Zhang Q., Cai K., Zhou H., Song Q., Han Z., Kang S., Li Y., Jiang Q., Zhang X., You J. (2025). Science.

[cit32] Chandrakar A., Khare A. (2025). Sol. Energy.

[cit33] Roy A., Ghosh A., Bhandari S., Sundaram S., Mallick T. K. (2020). Buildings.

[cit34] Lange S., Fett B., Kabakli Ö. S., Hähnel A., Adner D., Kroyer T., Bogati S., Schulze P. S. C., Herbig B., Hagendorf C., Sextl G., Mandel K. (2023). Sol. Energy Mater. Sol. Cells.

[cit35] Gutzler R., Kanevce A., Wahl T., Wessendorf C., Hempel W., Ahlswede E., Hariskos D., Paetel S. (2024). ACS Appl. Energy Mater..

[cit36] Nur-E-Alam M., Islam M. S., Abedin T., Islam M. A., Yap B. K., Kiong T. S., Das N., Rahman M. R., Khandaker M. U. (2025). Curr. Opin. Colloid Interface Sci..

[cit37] Suresh Kumar N., Chandra Babu Naidu K. (2021). Journal of Materiomics.

[cit38] Yesigat A., Geffe C. A., Abera N., Zhang Y., Zhang Q. (2026). J. Phys. Chem. Solids.

[cit39] Ke W., Stoumpos C. C., Kanatzidis M. G. (2019). Adv. Mater..

[cit40] Tang Z., Kuang X., Yu M., Chen J., Mao A. (2025). J. Mater. Chem. A.

[cit41] Zhu Z., Jiang X., Yu D., Yu N., Ning Z., Mi Q. (2022). ACS Energy Lett..

[cit42] Ma T., Zhao X., Yang X., Yan J., Luo D., Li M., Li X., Chen C., Song H., Tang J. (2024). Adv. Funct. Mater..

[cit43] Muslimawati R. M., Nursam N. M., Budiawan W., Mahyuddin M. H., Nuruddin A. (2025). J. Phys.:Conf. Ser..

[cit44] Shrivastav N., Aamir Hamid M., Madan J., Pandey R. (2024). Sol. Energy.

[cit45] Zhang Y., Shi L., Wang Z., Dai H., Hu Z., Zhou S., Chen H., Feng X., Zhu J., Sun Y., Liu W., Zhang Q. (2021). Sol. Energy.

[cit46] Mujahid A., Khan M. Y. H., Uddin M. M., Alhashmi Alamer F., Alsalmi O., Rasheduzzaman M., Hasan M. Z. (2025). RSC Adv..

[cit47] Srivastava S., Singh A. K., Kumar P., Pradhan B. (2022). J. Appl. Phys..

[cit48] Liao W., Zhao D., Yu Y., Grice C. R., Wang C., Cimaroli A. J., Schulz P., Meng W., Zhu K., Xiong R. G., Yan Y. (2016). Adv. Mater..

[cit49] Xu S., Zhang Y., Zhang Y., Zhou W., Huang X., Wang Z., Shi L., Zhang Q. (2026). Phys. Scr..

[cit50] Nie K., Liang Y., Chen X., Zhang Y., Wang J., Zhou C., Wang Z., Khalafallah D., Liu W., Zhang Q. (2025). Opt. Quantum Electron..

[cit51] Bhuvaneswari P., Sriramalakshmi P. (2025). Results Eng..

[cit52] Syeed M., Reza S., Ahammad A., Adnan M. M. R. (2026). J. Phys. Chem. Solids.

[cit53] Ahamed T., Uddin M. S., Ahammed T., Akteruzzaman M., Dipu M. H., Humayet Islam M., Ali Hossain M., Masum Billah M. (2026). Mater. Sci. Eng., B.

[cit54] Gham P. S., Priya A., Kumar R. (2026). J. Phys. Chem. Solids.

[cit55] Zhang Y., Xu S., Mu Z., Liu K., Chen J., Zhou C., Yao Y., Chen X., Shi L., Wang Z., Sun Y., Liu W., Zhang Q. (2022). Vacuum.

[cit56] Cheng S., Li B., Liu W., Zhang K., Zhang Y., He Z., Lin L., Sun S., Sun Y. (2020). Optik.

[cit57] Chirila A., Buecheler S., Pianezzi F., Bloesch P., Gretener C., Uhl A. R., Fella C., Kranz L., Perrenoud J., Seyrling S., Verma R., Nishiwaki S., Romanyuk Y. E., Bilger G., Tiwari A. N. (2011). Nat. Mater..

[cit58] Rombach F. M., Haque S. A., Macdonald T. J. (2021). Energy Environ. Sci..

[cit59] Singh P., Sengar B. S., Kumar A. (2024). J. Opt..

[cit60] Mattaparthi S., Bonal S. K. S., Tseng Z. L., Khosla R. (2025). Phys. Status Solidi A.

[cit61] Siddique M. A. F., Sayem Rahman A. S. M. (2024). Mater. Sci. Eng., B.

[cit62] Muslimawati R. M., Parlin M. S., Ramaputra S., Ariyanto F., Untoro A. A., Widianto E., Su'ait M. S., Nursam N. M., Wong L. H., Mahyuddin M. H., Yuliarto B., Nuruddin A. (2026). J. Phys. Chem. Solids.

[cit63] Abbas K. J., Bahrami A. (2024). Sol. Energy Mater. Sol. Cells.

[cit64] Shukla S., Sood M., Adeleye D., Peedle S., Kusch G., Dahliah D., Melchiorre M., Rignanese G.-M., Hautier G., Oliver R., Siebentritt S. (2021). Joule.

[cit65] Wang C., Hu Z., Liu Y., Cheng S., Yao Y., Zhang Y., Yang X., Zhou Z., Liu F., Zhang Y., Sun Y., Liu W. (2022). J. Mater. Sci.: Mater. Electron..

[cit66] Shi S., He Z., Fan Y., Liu Y., Zhang Y., Cheng S., Xu S., Zhang Y., Liu F., Zhou Z., Tang A., Sun Y., Liu W. (2019). Mater. Res. Express.

[cit67] Du Q., Li B., Shi S., Zhang K., Zhang Y., Cheng S., Zhou Z., Liu F., Sun S., Sun Y., Liu W. (2020). CrystEngComm.

